# Targeting the Gut Microbiota to Improve Dietary Protein Efficacy to Mitigate Sarcopenia

**DOI:** 10.3389/fnut.2021.656730

**Published:** 2021-06-21

**Authors:** Elena de Marco Castro, Caoileann H. Murphy, Helen M. Roche

**Affiliations:** ^1^Nutrigenomics Research Group, School of Public Health, Physiotherapy, and Sports Science, UCD Conway Institute, UCD Institute of Food and Health, University College Dublin, Dublin, Ireland; ^2^Teagasc Food Research Centre, Ashtown, Dublin, Ireland; ^3^Institute for Global Food Security, Queen's University Belfast, Belfast, United Kingdom

**Keywords:** protein digestibility, gut microbiota, sarcopenia, anabolic resistance, skeletal muscle, ageing, probiotic, leaky gut

## Abstract

Sarcopenia is characterised by the presence of diminished skeletal muscle mass and strength. It is relatively common in older adults as ageing is associated with anabolic resistance (a blunted muscle protein synthesis response to dietary protein consumption and resistance exercise). Therefore, interventions to counteract anabolic resistance may benefit sarcopenia prevention and are of utmost importance in the present ageing population. There is growing speculation that the gut microbiota may contribute to sarcopenia, as ageing is also associated with [1) dysbiosis, whereby the gut microbiota becomes less diverse, lacking in healthy butyrate-producing microorganisms and higher in pathogenic bacteria, and [2) loss of epithelial tight junction integrity in the lining of the gut, leading to increased gut permeability and higher metabolic endotoxemia. Animal data suggest that both elements may impact muscle physiology, but human data corroborating the causality of the association between gut microbiota and muscle mass and strength are lacking. Mechanisms wherein the gut microbiota may alter anabolic resistance include an attenuation of gut-derived low-grade inflammation and/or the increased digestibility of protein-containing foods and consequent higher aminoacidemia, both in favour of muscle protein synthesis. This review focuses on the putative links between the gut microbiota and skeletal muscle in the context of sarcopenia. We also address the issue of plant protein digestibility because plant proteins are increasingly important from an environmental sustainability perspective, yet they are less efficient at stimulating muscle protein synthesis than animal proteins.

## Introduction

Sarcopenia refers to the adverse muscle changes that accrue overtime, resulting in the loss of skeletal muscle quantity and quality (1). The estimated prevalence of sarcopenia in community-dwelling individuals aged over 50 years old is 1–29% and 14–33% in those living in long-term care facilities (2). Sarcopenia represents a threat to healthy ageing, as it can lead to difficulty in performing tasks of daily living, dependence, and frailty (3, 4). In terms of the underlying biology, skeletal muscle mass is largely regulated by muscle protein turnover, which comprises muscle protein synthesis (MPS) and breakdown (MPB). Protein turnover fluctuates daily in response to anabolic (i.e., dietary protein ingestion, exercise, and, to a lesser extent, hormonal stimulation) and catabolic stimuli (e.g., reduced amino acid and insulin concentrations due to fasting) (5–7). Dietary amino acids (AA) act as building blocks for *de novo* MPS, and indispensable AA (IAA), particularly leucine, act as potent anabolic signals, promoting translation initiation, largely *via* activation of mTORC1 (8). During basal, postabsorptive conditions, MPB rates exceed MPS rates, resulting in a negative net muscle protein balance (7, 9). In healthy young adults, this transient muscle protein loss is compensated by the increase in MPS, after protein ingestion due to hyperaminoacidemia (10), and decrease in MPB due to hyperinsulinemia (11). However, ageing is associated with a blunted MPS response to anabolic stimuli, known as “anabolic resistance,” which is one mechanism that can contribute to muscle loss overtime (8, 12).

Resistance exercise (13) and adequate nutritional intake, in particular energy and protein, are important factors contributing to sarcopenia prevention and treatment (14, 15). First, not only resistance exercise (16, 17) but also endurance exercise (18) sensitises the muscle to subsequent dietary protein ingestion, leading to a higher postprandial MPS response (13). In contrast, inactivity and muscle disuse, which tend to accompany ageing, show the opposite effect by desensitising the muscle to the anabolic effect of AA on MPS (19, 20). Second, higher protein intakes have been proposed to slow the loss of muscle mass in ageing (21) because higher protein (and leucine) intakes may compensate for anabolic resistance and induce an optimal postprandial MPS response (8, 15). Nevertheless, the interrelationship between dietary protein intake and long-term muscle and strength maintenance is complex. It is understood that low-protein intake may be a risk factor for sarcopenia since a greater amount of protein are required to stimulate MPS in the elderly (8, 12). However, many human dietary intervention studies have shown little or no impact of protein (22, 23), leucine (24–26), or IAA (27) supplementation on muscle mass and/or strength in non-exercising older adults. Therefore, resistance exercise, regulation of other nutrient sensors that modulate MPS aside from AA, and/or even greater amounts of protein supplementation/intake than those offered in this study may be essential tools to fight sarcopenia in healthy older adults. Given the age profile of the global population, we need to advance knowledge in relation to effective interventions to attenuate the age-related decline in muscle mass and function.

## Evidence for the Gut-Muscle Axis

Over the last two decades, there has been an exponential interest in the role of gut microbiota in health and disease (28). To this end, this review explores if and how this may play a role in relation to the gut-muscle axis within the context of sarcopenia. Before examining the emerging evidence in relation to a potential role of the gut microbiota in muscle mass and function (29–31), it is important to acknowledge the wealth of knowledge in relation to factors that affect anabolic resistance (8, 12, 32) and sarcopenia. Mechanisms involved in sarcopenia include poor nutrition (21), physical inactivity (19, 20), changes in hormone levels and sensitivity, especially insulin (33, 34), mitochondrial dysfunction (35), aberrant intermuscular and intramuscular fat deposition (36), and chronic low-grade inflammation (37, 38) [discussed here (39)].

From the microbiota perspective, the interrelationships between gut and muscle are not firmly established; nevertheless, there are some interesting developing perspectives. Backhed et al. (30) laid the foundation for recent research on the gut-muscle axis, showing that germ-free mice exhibit a lean phenotype, even on high-calorie, high-fat diets. Subsequent studies examining the skeletal muscle of germ-free (free of all microorganisms) vs. pathogen-free mice (free of pathogenic microorganisms) provided key insights into the gut-muscle axis (29). Compared with pathogen-free mice, germ-free mice had reduced skeletal muscle mass, strength, and IGF-1 local expression and increased local expression of genes associated with muscle atrophy (*FoxO, Atrogin-1, Murf-1*, and *MyoD*) (29). The explanation of the authors for the observed muscle mass reduction in germ-free mice is an increase in MPB, rather than a significant reduction in MPS, as that activation of Akt-mTOR-S6k was unaffected in germ-free mice (29). Muscle protein turnover was not directly measured. It is important to note that, in humans, changes in MPS due to diet-mediated hyperaminoacidemia and resistance exercise, rather than alterations in MPB, are better understood and believed to largely determine net protein balance and, ultimately, muscle size in nonpathogenic states (40, 41). Gut microbiota transplantation from pathogen-free mice into germ-free mice restored skeletal muscle mass, reduced muscle atrophy markers, improved oxidative metabolic capacity of the muscle, and elevated *Rapsyn* and *Lrp4* expression, both of which are essential for neuromuscular junction maintenance (29, 42). Finally, they treated germ-free mice with a short-chain fatty acid (SCFA) blend of acetate, butyrate, and propionate, similar to what is produced by a healthy microbiota upon polysaccharides fermentation and partially reversed the skeletal muscle impairments and improved muscle strength (29). In addition, antibiotic-induced depletion of microbiota inmurine studies shows a reduction in muscle mass to a body mass ratio in comparison to the microbiota-containing control (43–45). However, restoring their microbiota led to an increase of the muscle mass to the body mass ratio. We acknowledge the difficulty in translating these data from mice to humans (46) and that germ- and pathogen-free models are not a feasible study design to understand the effect of the gut microbiota on muscle in humans; however, germ-free mice models demonstrate the relevance of the gut in muscle mass and function *in vivo*.

## Ageing and the Gut Microbiota—Insights From Human Studies

The gut microbiota of a typical older person displays reduced species richness and higher interindividual variability, together with less beneficial butyrate-producing bacteria and tight junction integrity, and a greater prevalence of pathogenic gram-negative bacteria (47–49). The fact that older people, especially the older and frail subjects (31, 50), have a distinct gut microbiota composition leads us and others (31, 51–56) to hypothesise the involvement of the gut microbiota in sarcopenia development.

Claesson et al. (31) carried out a cross-sectional study in 178 individuals aged 78 ± 8 years old and living in three different settings (community dwelling, short-term rehabilitation hospital care, and long-term residential care). They showed that the gut microbiota of the participant was clustered by living setting and was related to dietary intake (31). The “unfavourable microbiota profile,” displaying lower diversity in the long-term residential care individuals, was correlated to frailty, comorbidities, poor nutritional status, and inflammation markers (31). Compared with community dwellers, short-term stay seniors showed a lower frequency of microbial genes for SCFA and higher serum markers of inflammation (31). The differences in dietary intake, medication use, and clinical status between the subgroups had an effect on modulating the gut microbiota (52, 57, 58) and may have acted as confounding variables that contributed to the associations observed between health and gut microbiota composition in the study (31). Other studies also suggest that physical frailty is inversely related to gut microbiota biodiversity and the relative abundance of a number of key taxa (31, 50, 52, 59–63). However, even if these studies reduced or excluded confounding factors, it is not possible in observational study designs to establish a cause–effect relationship between a specific gut microbiota composition or taxa and healthy ageing or longevity. In a recent attempt to evaluate a causative role for the gut microbiota in body composition and strength, germ-free mice were colonised with the gut microbiota of high- and low-functioning older adults (64). Compellingly, muscle strength, but not lean mass or endurance, was significantly higher in mice colonised with microbiota from high- vs. low-functioning older adults (64).

## Probiotic and the Gut Microbiota

**Figure 1:**
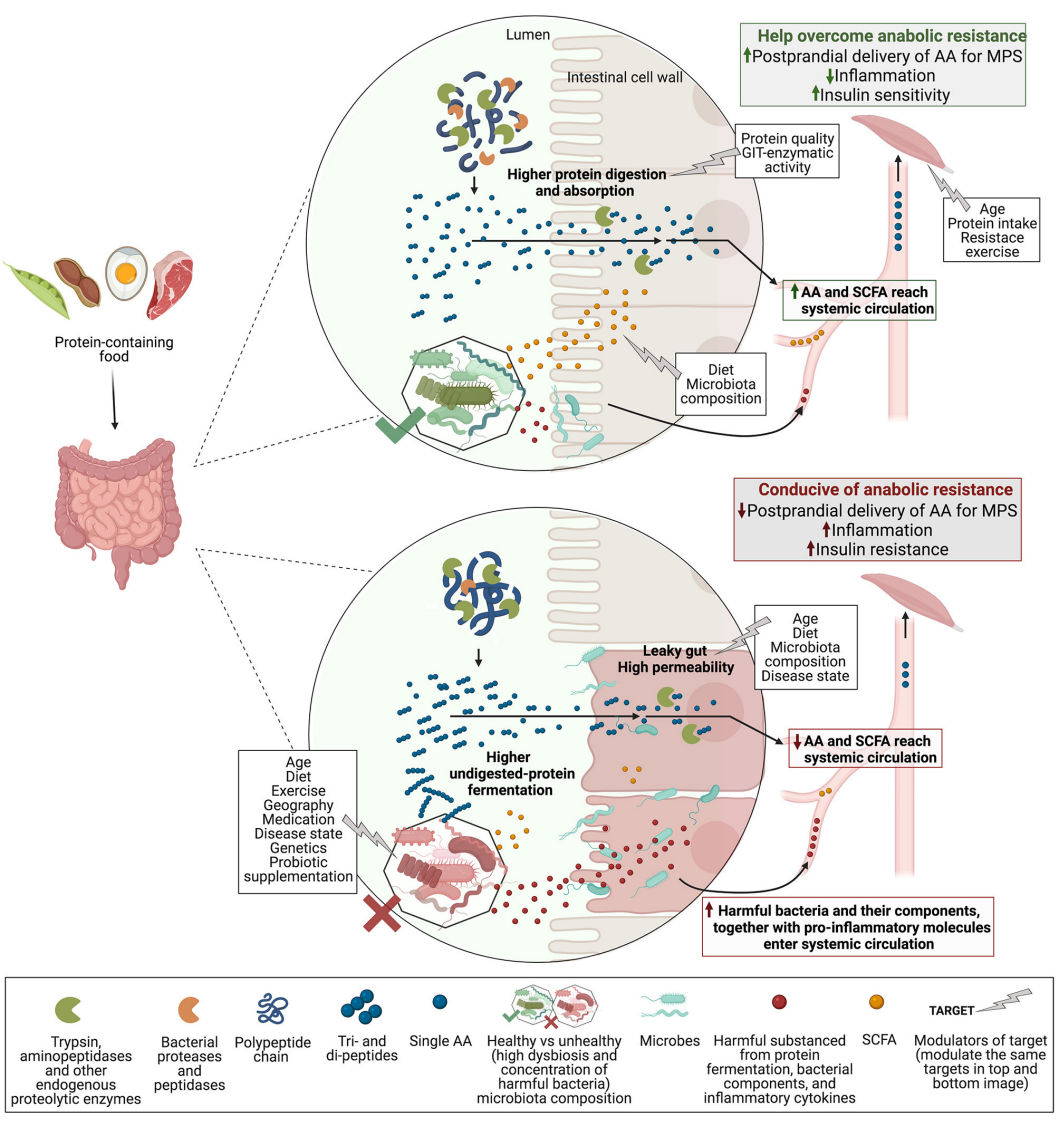
Proposed the gut microbiota role in protein digestion and absorption, and related MPS response. Polypeptides from protein-containing foods enter the duodenum and are cleaved by enzymes into di- and tripeptides, and some single AA, which are absorbed by enterocytes (intestinal cell wall). Aminopeptidases of enterocytes cleave di- and tripeptides into single AA that can now enter systemic circulation after first-pass splanchnic retention. In the lumen, enzymes of gut microbes or probiotics may aid with the polypeptide and shorter peptides cleavage to increase AA delivery to the bloodstream. Undigested peptides reach the colon and are fermented by the gut microbiota to release molecules like ammonia and hydrogen sulphide (harmful), and SCFA (in lesser amounts, SCFAs are mainly ferments of undigested carbohydrates). A healthy gut microbiota composition (high concentration of beneficial microbes and diversity) may release higher amounts of SCFA and is better able to control the translocation of harmful substances from the lumen into circulation. SCFA can improve gut permeability and may positively modulate muscle biology. Reduced translocation of proinflammatory molecules is linked to lower systemic inflammation and may positively influence insulin sensitivity in the muscle. Opposingly, unhealthy gut microbiota is associated with a leaky gut, which is less able to regulate the harmful translocation into the bloodstream of microbes (e.g., *Bacteroides sp*.) and their components (e.g., LPS), as well as proinflammatory cytokines, all likely to be found in an “unhealthy” GIT. This leads to low-grade chronic systemic inflammation that may contribute to insulin and anabolic resistance in the muscle. Therefore, healthy gut microbiota may improve protein digestion and absorption by increasing peptide cleavage, and, in addition, promote SCFA production and reduce protein fermentation and the “leaky gut.” As a result, more AA and SCFA, and less gut-derived harmful molecules enter systemic circulation, increasing the postprandial delivery of AA to the muscle and reducing systemic and local inflammation, both in favour of MPS. Abbreviations: MPS, muscle protein synthesis; AA, amino acids; SCFA, short-chain fatty acids; LPS, lipopolysaccharide; GIT, gastrointestinal tract—created with BioRender.com.

Probiotics can be defined as “live microorganisms that when administered in adequate amounts confer a health benefit to the host” (65). Recent evidence points towards probiotic supplementation as a plausible nutrition intervention to improve muscle mass and/or function (66–73) and to help prevent sarcopenia. To our knowledge, there is no evidence in relation to the impact of probiotic supplementation on MPS, but a possible role may be a probiotic-directed improvement of protein digestibility, gut permeability, and SCFA production. While these mechanisms do not inherently alter the protein composition of the source (e.g., IAA or leucine content), better digestion may increase postprandial aminoacidemia, and improved gut permeability may reduce chronic inflammation, both in favour of MPS (Figure 1).

## Probiotic and the Gut-Muscle Axis

Bindels et al. (66) led one of the pioneer studies on gut microbiota modulation as a way to alter muscle in leukemic mice. Oral probiotic supplementation (*Lactobacillus reuteri* and *Lactobacillus gasseri*, but not *Lactobacillus acidophilus*) restored gut microbiota health from a baseline status of dysbiosis, reduced serum levels of pro-inflammatory cytokines, and increased muscle mass (66). In another murine cancer model, *L. reuteri* supplementation reduced systemic inflammation and preserved muscle mass (67). In healthy young mice without systemic inflammation, probiotic *Lactobacillus plantarum* supplementation not only improved lean mass but also muscle function (68). A series of recent reviews (74–78) and original studies (69–73) have investigated the impact of probiotic supplementation on lean mass and physical performance in humans. After probiotic supplementation, some studies in athletes (predominately males) showed improvements in muscle strength, power, and exercise recovery (69–72), but none observed significant alterations in body lean mass (69–73). The extent to which probiotic supplementation is able to alter the intestinal gut microbiota is under debate (79, 80). However, a proprietary form of *Bacillus coagulans* has demonstrated an advantageous spore-forming ability to survive the harsh condition of the stomach, which enables it to create a healthier gut microbiota composition in the elderly (81) and aid with the digestion of plant-based proteins in a validated *in vitro* model of the stomach and small intestine (82).

In humans, only two studies, to our knowledge, have investigated the effect of probiotic supplementation on aminoacidemia, following pea (83) and milk (84) protein ingestion. After 2 weeks of protein supplementation with or without added probiotics, the participants reported to the laboratory, and blood samples were taken in the fasted state and following the ingestion of their respective supplement. In one study, 15 physically active young men consumed isolated pea protein with or without probiotic *Lactobacillus paracasei* (83). In the other study, 30 young males and females ingested milk protein concentrate with or without *B. coagulans* (84). Both studies used postprandial maximum systemic AA concentration (C_max_) and area under the curve (AUC) as proxy measurements for AA digestion and absorption. C_max_ and AUC were higher after protein and probiotic co-ingestion in comparison with protein alone in both studies (83, 84). Such an increase in aminoacidemia may improve the muscle anabolic response to dietary protein (70), although that outcome was not determined.

## Putative Mechanism 1: Probiotics and Protein Digestibility

In addition to total protein intake, the quality of the protein consumed in the diet is another important consideration for muscle mass preservation with age. Plant proteins are increasingly important from an environmental sustainability perspective (85), yet they are less efficient at stimulating muscle protein synthesis than animal proteins at the same protein dose (86). Plant vs. animal proteins show lower digestibility (45–80% vs. >90%, respectively) in both young and old individuals due to high levels of insoluble fibres and/or anti-nutritional factors (87–89). Anti-nutritional factors present endogenously, like trypsin inhibitors in grain legumes or tannins in cereals (90), or are formed during excessive heat and/or alkaline processing, like Maillard compounds in milk, and may reduce protein bioavailability (91, 92). Contrarily, the processing of dietary plant proteins (gentle heating, soaking, germination, and fermentation) may have a positive effect on digestibility (93–95). This is important for plant proteins because poor digestibility results in lower AA absorption and thus reduced AA availability for MPS (96). Although the difference between protein digestion and absorption kinetics in older vs. young individuals does not appear to be the limiting factor for the observed difference in MPS (97, 98), improving plant protein digestibility would enhance the delivery of AA to circulation (and of special interest IAA). This has already been identified in young healthy humans for pea protein (83) and milk protein (84) with probiotic co-ingestion, as mentioned above. An explanation for this could be the release of proteases and peptidases by the probiotic to facilitate protein digestion in the small intestine, although this hypothesis would have been better supported if the protein had been ingested in its raw form (as part of the intact food source; peas and milk) and not as a protein isolate. This is particularly relevant in older individuals as they need more protein per meal to maximise the postprandial MPS response (99) and, thus, to preserve muscle mass with age (100). An improvement in protein digestibility would imply that older people could reduce the net amount of protein consumption per meal for the same MPS effect, which is crucial, given their reduced appetite (101).

## Putative Mechanism 2: Probiotics and the Leaky Gut

The intestinal barrier is the key to maintaining gut integrity, preventing leakage of bacterial cells and/or their proinflammatory toxins into the bloodstream (102). The “leaky gut” hypothesis proposes that as a result of intestinal barrier breakdown, its permeability increases and is less able to regulate the translocating of harmful substances, triggering the immune system and inflammatory responses (102, 103). In this review, we speculate that the leaky gut may be associated with sarcopenia since low-grade chronic inflammation in older adults is one of the factors believed to contribute to anabolic resistance and thus sarcopenia development (38).

Rodent studies indicate that ageing is associated with a leaky gut *via* gut-derived metabolic endotoxemia and low-grade chronic inflammation (104–106). Poor mucosal barrier function and increased inflammation have also been reported in aged vs. young monkeys (103, 107). Qi et al. (108) measured zonulin, a physiologic regulator of intestinal permeability (109), in healthy young (18–30 years, *n* = 19) and old (≥70 years, *n* = 18) adults. Serum zonulin concentrations were significantly higher in older vs. younger adults, were positively associated with proinflammatory cytokine levels (TNF-α and IL-6), and were negatively associated with physical activity levels and skeletal muscle strength (108). Furthermore, the serum microbiota of young (20–35 years, *n* = 24) vs. old (60–75 years *n* = 24) individuals differs and is linked to markers of age-related systemic inflammation (110). While these data support the role of a leaky gut in age-directed inflammation and frailty, data from Valentini et al. (111) concluded that small intestinal barrier integrity is not altered in healthy ageing. However, it supported that low-grade chronic inflammation, which is common in older adults (38, 112), compromises intestinal barrier permeability (111). Therefore, one may question whether age-directed low-grade chronic inflammation is the cause or the result of gut barrier breakdown (113, 114).

It could be hypothesised that, irrespective of age, “fixing” a leaky gut may enhance muscle function by reducing the amount of detrimental microbial products (e.g., LPS and indoxyl sulphate) that access systemic circulation. These harmful substances are known to trigger low-grade systemic inflammation, insulin resistance, and glucose intolerance in murine skeletal muscle (115, 116). They have also been reported to increase NFκB activity and JNK phosphorylation, blunt AMPK phosphorylation in skeletal muscle tissue of patients with type 2 diabetes (117), and reduce insulin sensitivity in LPS-treated human muscle cells (118).

Emerging evidence points towards a potential role for probiotic-mediated alleviation of the leaky gut. Probiotic supplementation improved markers of an intestinal barrier and inflammation in trained men under exercise stress in a randomised clinical trial (119), in rodents (120, 121), and in a porcine intestinal epithelial cell line (IPEC-J2) (122). However, a systematic review from 2013 that included human studies measuring parameters of epithelial barrier function had evidence for and against probiotic supplementation (123). Note that the study population and probiotics employed were high in heterogeneity. Therefore, more studies need to evaluate the effect of microbiota modulation on the leaky gut and resulting microbiota-derived inflammatory markers in circulation and their effect on skeletal human muscle.

## Putative Mechanism 3: Probiotics and Muscle Nutrient Sensors

Another putative link between gut microbiota modulation and the muscle could be regulated by muscle nutrient sensors, which are gut microbiota by-products, mainly SCFAs (29), could alter muscle biology [reviewed here (124, 125)]. Probiotic bacteria are known producers of SCFAs (45, 126, 127). Emerging evidence from young germ-free mice fed with SCFAs shows an increase in skeletal muscle mass and strength compared with the untreated control (29). Also, sodium butyrate-supplemented high-fat diet-fed young mice exhibited a reduction in skeletal muscle insulin resistance vs. their non-supplemented counterparts (128). Similarly, aged mice under a 10-month sodium butyrate-supplemented diet treatment showed an increase in muscle mass to body mass ratio when compared with the non-supplemented control (129). Interestingly, SCFA-producing bacteria include *Barnesiella* and *Prevotellaceae* and are both found in higher concentration in high- vs. low-functioning older adults, characterised based on the percentage of body lean mass and physical functioning (64). These studies show a strong association between SCFA and muscle mass and strength; however, whether this link is casual in humans and its mechanism is not known.

## Conclusion and Future Perspectives

Ageing is often associated with a reduction in muscle mass and function together with a reduction in abundance, resilience, and diversity of the gut microbiota (31, 50, 61, 62). While emerging evidence supports a putative link between the gut and the muscle that could be a potential target for the prevention and treatment of sarcopenia, a causal relationship between the gut microbiota and muscle protein synthesis has not yet been established in humans, albeit evidence from murine models is strong (29). However, the data examined in this review may underpin the hypothesis that a healthy and diverse gut microbiota in an elderly cohort, potentially modulated *via* probiotic supplementation, may improve age-associated muscle decline, mechanistically, (1) by improving the intestinal cell wall integrity, thus reducing metabolic endotoxemia and consequential inflammation linked to insulin resistance and anabolic resistance (also potentially modulated by short-chain fatty acids), and/or (2) by improving dietary protein digestion and absorption *via* gut-bacterial enzymatic activity, leading to enhanced amino acid availability for *de novo* protein synthesis. These points are relevant in the context of healthy ageing in the present ever-growing older population (130), where enhancing and preserving physical strength, mobility, and independence are of utmost importance. The review also highlights the need for a greater understanding in relation to the area of nutrition that deals with sarcopenia prevention and treatment based on protein supplementation research disagreements on muscle mass and strength in older adults. Within this research gap, we acknowledge that the plant protein industry is booming, stemmed from environmental, ethical, and health interests (131). Even though plant protein may be less effective for preserving muscle mass in ageing, this presents a research opportunity to clearly define the efficacy of plant proteins on *de novo* protein synthesis in humans, which may or may not be affected by the gut microbiota. Overall, improving the digestibility and absorption of alternative protein sources and their ability to stimulate muscle protein synthesis will ease the environmental and nutritional challenges of the future global population while also favouring functional ageing.

## Author Contributions

EM drafted the manuscript with advice from CM and HR. CM and HR provided critical revisions. All the authors approved the final version of the manuscript.

## Conflict of Interest

EM is in receipt of a Ph.D. studentship co-funded by the Irish Research Council and Kerry. The remaining authors declare that the research was conducted in the absence of any commercial or financial relationships that could be construed as a potential conflict of interest.
